# Risk of Bias in Randomized Clinical Trials Comparing Transcatheter and Surgical Aortic Valve Replacement: A Systematic Review and Meta-analysis

**DOI:** 10.1001/jamanetworkopen.2022.49321

**Published:** 2023-01-03

**Authors:** Fabio Barili, James M. Brophy, Daniele Ronco, Patrick O. Myers, Miguel Sousa Uva, Rui M. S. Almeida, Mateo Marin-Cuartas, Amedeo Anselmi, Jacques Tomasi, Jean-Philippe Verhoye, Francesco Musumeci, John Mandrola, Sanjay Kaul, Stefania Papatheodorou, Alessandro Parolari

**Affiliations:** 1 Department of Epidemiology, Harvard T. H. Chan School of Public Health, Boston, Massachusetts; 2 Department of Cardiac Surgery, S. Croce Hospital, Cuneo, Italy; 3 Department of Medicine, McGill Health University Center, Montreal, Quebec, Canada; 4 Department of University Cardiac Surgery, IRCCS Policlinico San Donato, Milan, Italy; 5 Division of Cardiac Surgery, CHUV–Lausanne University Hospital, Lausanne, Switzerland; 6 Department of Cardiac Surgery, Hospital Santa Cruz, Carnaxide, Portugal; 7 Department of Cardiac Surgery and Physiology, Porto University Medical School, Porto, Portugal; 8 University Center Assis Gurgacz Foundation, Cascavel, Paraná, Brazil; 9 University Department of Cardiac Surgery, Leipzig Heart Center, Leipzig, Germany; 10 Department of Thoracic and Cardiovascular Surgery, University Hospital of Rennes, Rennes, France; 11 Department of Cardiac Surgery and Heart Transplantation, San Camillo Forlanini Hospital, Rome, Italy; 12 Baptist Health Louisville, Louisville, Kentucky; 13 Department of Cardiology, Cedars-Sinai Medical Center, Los Angeles, California

## Abstract

**Importance:**

Recent European Society of Cardiology/European Association for Cardio-Thoracic Surgery (ESC/EACTS) guidelines highlighted some concerns about the randomized clinical trials (RCTs) comparing transcatheter aortic valve implantation (TAVI) and surgical aortic valve replacement (SAVR) for aortic stenosis. Quantification of these biases has not been previously performed.

**Objective:**

To assess whether randomization protects RCTs comparing TAVI and SAVR from biases other than nonrandom allocation.

**Data Sources:**

A systematic review of the literature between January 1, 2007, and June 6, 2022, on MEDLINE, Embase, and Cochrane Central Register of Controlled Trials was performed. Specialist websites were also checked for unpublished data.

**Study Selection:**

The study included RCTs with random allocation to TAVI or SAVR with a maximum 5-year follow-up.

**Data Extraction and Synthesis:**

Data extraction was performed by 2 independent investigators following the PRISMA guidelines. A random-effects meta-analysis was used for quantifying pooled rates and differential rates between treatments of deviation from random assigned treatment (DAT), loss to follow-up, and receipt of additional treatments.

**Main Outcomes and Measures:**

The primary outcomes were the proportion of DAT, loss to follow-up, and patients who were provided additional treatments and myocardial revascularization, together with their ratio between treatments. The measures were the pooled overall proportion of the primary outcomes and the risk ratio (RR) in the TAVI vs SAVR groups.

**Results:**

The search identified 8 eligible trials including 8849 participants randomly assigned to undergo TAVI (n = 4458) or SAVR (n = 4391). The pooled proportion of DAT among the sample was 4.2% (95% CI, 3.0%-5.6%), favoring TAVI (pooled RR vs SAVR, 0.16; 95% CI, 0.08-0.36; *P* < .001). The pooled proportion of loss to follow-up was 4.8% (95% CI, 2.7%-7.3%). Meta-regression showed a significant association between the proportion of participants lost to follow-up and follow-up time (slope, 0.042; 95% CI, 0.017-0.066; *P* < .001). There was an imbalance of loss to follow-up favoring TAVI (RR, 0.39; 95% CI, 0.28-0.55; *P* < .001). The pooled proportion of patients who had additional procedures was 10.4% (95% CI, 4.4%-18.5%): 4.6% (95% CI, 1.5%-9.3%) in the TAVI group and 16.5% (95% CI, 7.5%-28.1%) in the SAVR group (RR, 0.27; 95% CI, 0.15-0.50; *P* < .001). The imbalance between groups also favored TAVI for additional myocardial revascularization (RR, 0.40; 95% CI, 0.24-0.68; *P* < .001).

**Conclusions and Relevance:**

This study suggests that, in RCTs comparing TAVI vs SAVR, there are substantial proportions of DAT, loss to follow-up, and additional procedures together with systematic selective imbalance in the same direction characterized by significantly lower proportions of patients undergoing TAVI that might affect internal validity.

## Key Points

**Question:**

Does randomization protect randomized clinical trials (RCTs) comparing transcatheter aortic valve implantation (TAVI) and surgical aortic valve replacement (SAVR) from biases other than nonrandom allocation?

**Findings:**

This systematic review and meta-analysis of 8 RCTs including 8849 participants and comparing TAVI vs SAVR found substantial overall proportions of deviation from assigned treatment, loss to follow-up, additional procedures, and additional myocardial revascularization together with a systematic selective imbalance in the same direction characterized by significantly lower proportions among participants undergoing TAVI.

**Meaning:**

This study suggests that RCTs comparing TAVI and SAVR show serious methodological imbalances with a common selective pattern, and should be considered at high risk of performance and attrition bias that may affect internal validity.

## Teaser

This systematic review and meta-analysis assesses whether randomization protects randomized clinical trials comparing transcatheter aortic valve implantation and surgical aortic valve replacement from biases other than nonrandom allocation.

## Introduction

The literature on the comparison between transcatheter and surgical approaches for aortic valve stenosis comprises 7 randomized clinical trials (RCTs) conducted since 2007 in progressively lower-risk populations that demonstrated noninferiority and, in some cases, superiority of transcatheter aortic valve implantation (TAVI) compared with the standard of care.^1,2,3,4,5,6,7,8,9,10,11,12,13,14^ These RCTs form the basis of guidelines on the management of heart valve disease; nevertheless, several concerns on study design remain.^15,16,17,18^ Although randomization allows control for confounding on admission, exclusion after randomization and deviations from protocol related to additional procedures can still introduce bias.^17,18^ Exclusion after randomization first occurs when participants do not adhere to or receive the assigned treatment and can be summarized as the difference between intention-to-treat (ITT) and as-treated cohorts. This difference is also known as deviations from random assigned treatment (DAT).^19^ The erosion of the unbiased ITT groups generated by nonrandom loss of participants produces biases related to the violation of the principle of randomization, particularly if nonrandom loss of participants is selectively represented in the treatments.^19,20,21^ In RCTs designed for evaluating outcomes at follow-up, the main source of exclusion is loss to follow-up, as participants might withdraw from the study or might not be located.^19,22,23^ Each loss impairs internal validity, especially if it is differential among treatment groups. The imbalance related to patients’ exclusion for nonadherence to treatments or loss to follow-up could lead to attrition bias,^22,23^ which might undermine the advantages of randomization, thereby challenging the comparability of the treatment groups.

Another major issue that emerged in the 2021 European Society of Cardiology/European Association for Cardio-Thoracic Surgery (ESC/EACTS) guidelines’^18^ quality assessment by version 2 of the Cochrane risk-of-bias tool for randomized trials (RoB 2 tool) is the risk of performance bias that occurs if additional treatments are provided preferentially to a single group, potentially leading to biased outcomes.^22,23^ An imbalance in concomitant procedures (revascularization and other additional procedures) between patients who underwent TAVI and those who underwent surgical aortic valve replacement (SAVR) is present in almost all the RCTs. Although some of these procedures cannot be defined as protocol deviation because they are allowed according to the protocol, each treatment other than isolated aortic valve replacement may affect short- and long-term outcomes, and differential rates of additional procedures may selectively affect the results.

Although the assessment of methodological quality of RCTs by the RoB 2 tool is mostly qualitative,^24^ we performed a quantitative assessment of DAT, loss to follow-up, and receipt of additional treatments for the TAVI vs SAVR trials and an evaluation of their differential rates between treatments.

## Methods

### Search Strategy and Selection Criteria

This meta-analysis study is exempt from ethics approval as we collected and synthesized data published from previous clinical trials in which informed consent had already been obtained by the trial investigators. The study protocol adhered to the Preferred Reporting Items for Systematic Reviews and Meta-analyses (PRISMA) reporting guideline.^25^ The protocol has been registered in PROSPERO.^26^

We searched publications from MEDLINE, Embase, and the Cochrane Central Register of Controlled Trials from January 1, 2007, to June 6, 2022. We also checked websites^27,28,29,30,31^ for unpublished data. The search algorithm has been published^16^ and is detailed in the eTable in Supplement 1. We included RCTs with random allocation to undergo TAVI or SAVR with a maximal follow-up of 5 years.

### Outcomes Measures

The primary outcomes were the proportion of DAT, loss to follow-up, patients who underwent additional interventions, and additional myocardial revascularization. DAT was calculated as the difference between the ITT and as-treated cohorts. Loss to follow-up was extracted at all available follow-up times.

Proportion meta-analysis was used to calculate the pooled overall proportion of the primary outcomes. Risk ratio (RR) was used as effect size for evaluating differential rates of the primary outcomes between TAVI and SAVR (with SAVR as the control group).

### Data Extraction

Two independent investigators (F.B. and D.R.) identified trials that fulfilled the prespecified inclusion criteria. Eligible trials were then reviewed in duplicate and disagreement was resolved by a third investigator (A.P.). Extracted data from the text and appendixes of eligible trials were trial characteristics, patients’ baseline data and comorbidities, device type, and implantation access.

### Risk of Bias and Quality Assessment

This meta-analysis quantitatively evaluated 2 domains of the RoB 2 tool. The risk of bias among trials included in the ESC/EACTS 2021 Guidelines for Management of Heart Valve Disease by the 4 delegates^18^ was then updated by 2 researchers (F.B. and A.P.), with a third researcher (A.A.) designated to resolve potential disagreements using the RoB 2 tool,^24^ incorporating information provided by outcomes of this meta-analysis.

### Statistical Analysis

Random-effects proportion meta-analysis was used to estimate the pooled overall proportions of the primary outcomes.^32^ The between-study variance was estimated with the Hartung-Knapp-Sidik-Jonkman method, while the Cochran *Q* test and the *I*^2^ statistics were applied to evaluate between-study heterogeneity.

Random-effects meta-analysis was used to pool the RR of primary outcomes in the TAVI vs SAVR groups.^33^ Between-study variance was estimated with the Hartung-Knapp-Sidik-Jonkman method, while the Cochran *Q* test and the *I*^2^ statistics were applied to evaluate between-study heterogeneity. The association between loss to follow-up and follow-up time was explored with random-effects meta-regression. Analyses were performed with R, version 4.2.0 (R Group for Statistical Computing).^34^ All *P* values were from 2-sided tests and results were deemed statistically significant at *P* < .05.

## Results

### Trials Characteristics

Nine trials were checked for further assessment.^1,2,3,4,5,6,7,8,10,11,12,13,15,16,35,36^ The STACCATO (Transapical Transcatheter Aortic Valve Implantation vs Surgical Aortic Valve Replacement in Operable Elderly Patients With Aortic Stenosis) trial^35^ was excluded as it was prematurely terminated by the data safety monitoring board. The remaining 8 trials (PARTNER [Placement of Aortic Transcatheter Valve Trial] 1A, PARTNER 2A, PARTNER 3, CoreValve US Pivotal High Risk Trial, SURTAVI [Surgical Replacement and Transcatheter Aortic Valve Implantation] Trial, Evolut Low Risk Trial, NOTION [Nordic Aortic Valve Intervention] Trial, and UK TAVI [Transcatether Aortic Valve Implantation] Trial) fulfilled the prespecified inclusion criteria and were included in the meta-analysis (eFigure 1 in Supplement 1).^1,2,3,4,5,6,7,8,10,11,12,13,15,16,36^

Baseline characteristics of the included RCTs are detailed in the eTable in Supplement 2. All studies were multicenter RCTs. The primary analysis was ITT analysis in PARTNER 1A, PARTNER 2A, and the UK TAVI Trial; modified ITT (patients who had undergone randomization and an attempted procedure) analysis in SURTAVI; and as-treated analysis in PARTNER 3, CoreValve US Pivotal High Risk Trial, Evolut Low Risk Trial, and NOTION Trial. Overall, 8849 patients were randomly assigned to undergo TAVI (n = 4458) or SAVR (n = 4391). In the 8 trials, both balloon-expanding and self-expanding TAVI devices were evaluated. Different approaches were used for TAVI; however, the most common access was transfemoral.

### Analysis of DAT

The pooled proportion of DAT was 4.2% (95% CI, 3.0%-5.6%) (eFigure 2 in Supplement 1), with significant heterogeneity among the studies (τ^2^ = 0.0017; *Q* test *P* < .001; *I*^2^ = 86%). DAT showed a selective pattern, being 6.2-fold lower in the TAVI group compared with the SAVR group (pooled RR, 0.16; 95% CI, 0.08-0.36; *P* < .001) (eFigure 3 in Supplement 1).

**Figure 1.**
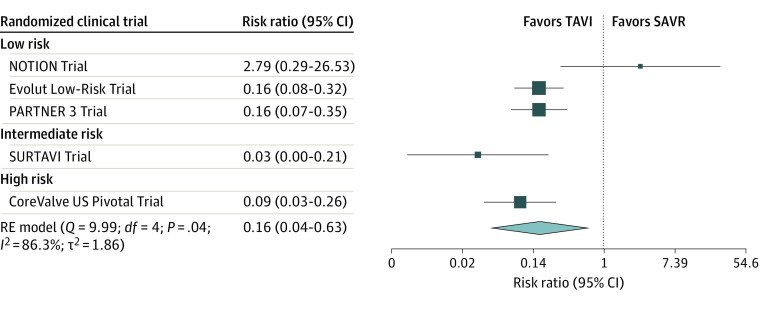
Forest Plot of Risk Ratio of Deviation From Assigned Treatment (DAT) in Transcatheter Aortic Valve Implantation (TAVI) vs Surgical Aortic Valve Replacement (SAVR) (Selective DAT) in Randomized Clinical Trials That Performed As-Treated or Modified Intention-to-Treat Analysis There is a selective pattern characterized by a lower proportion of DAT in the TAVI group resulting in a 6.2-fold lower proportion of DAT in the TAVI group compared with the SAVR group. Only the NOTION (Nordic Aortic Valve Intervention) Trial does not show a selective DAT. The size of the solid squares is proportional to the weight of each study, the horizontal bars indicate the 95% CI for each study, and the diamond represents the pooled estimate with 95% CI. PARTNER 3 indicates Placement of Aortic Transcatheter Valve Trial 3; RE, random effect; and SURTAVI, Surgical Replacement and Transcatheter Aortic Valve Implantation.

The pooled proportion of DAT in the subgroup of 5 studies that did not perform ITT analysis was 3.6% (95% CI, 2.1%-5.5%) (eFigure 4 in Supplement 1). Also, this subset showed a differential rate of DAT, with a 6.2-fold lower proportion of DAT in the TAVI group compared with SAVR group (RR, 0.16; 95% CI, 0.04-0.63; *P* = .008) (Figure 1). Only the NOTION Trial did not show a selective DAT.

### Analysis of Loss to Follow-up

**Figure 2.**
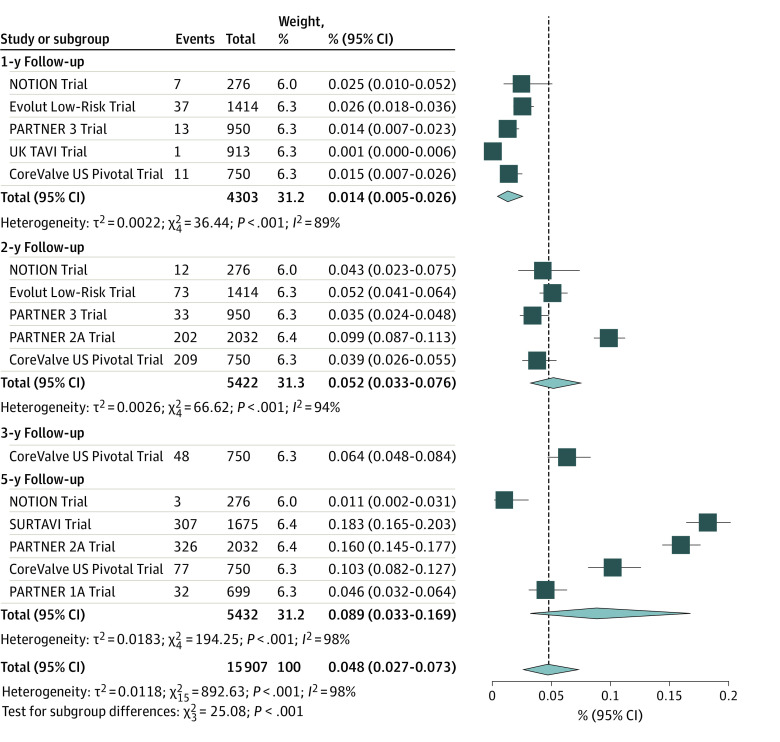
Forest Plot Presenting the Proportion Meta-analysis of Patients Lost to Follow-up per Year of Follow-up On average, 4.8% of patients at risk were missing at each follow-up time, with a progressive increase of proportion of patients lost to follow-up with ongoing follow-up, as shown by the increasing rates among subgroups of follow-up time. Diamonds indicate the pooled proportions with 95% CIs; the vertical dashed line represents the pooled overall proportion. NOTION indicates Nordic Aortic Valve Intervention Trial; PARTNER, Placement of Aortic Transcatheter Valve Trial; SURTAVI, Surgical Replacement and Transcatheter Aortic Valve Implantation; and TAVI, transcatheter aortic valve implantation.

**Figure 3.**
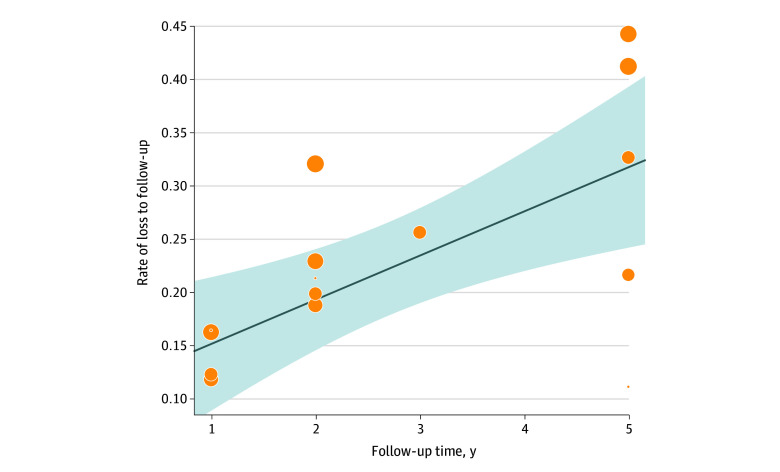
Meta-regression of Association Between Loss to Follow-up and Follow-up Time There is a significant association between proportion of patients lost to follow-up and follow-up time. Shaded area indicates 95% CI; diagonal line indicates the linear association between loss to follow-up and follow-up time; circles indicate the rates of loss to follow-up at follow-up time for each randomized clinical trial; and circle size indicates the weight of single data.

Figure 2 shows a forest plot presenting the proportional meta-analysis of loss to follow-up by year of follow-up in the 8 included RCTs. The pooled proportion of loss to follow-up was 4.8% (95% CI, 2.7%-7.3%), indicating that, on average, 4.8% of patients at risk were missing at each follow-up time. A subgroup analysis on different follow-up times demonstrated that the prevalence of loss to follow-up increased concordantly with follow-up time, increasing from 1.4% (95% CI, 0.5%-2.6%) at 1 year to 8.9% (95% CI, 3.3%-16.9%) at 5 years. Meta-regression confirmed a significant association between the proportion of participants lost to follow-up and follow-up time (slope, 0.042; 95% CI, 0.017-0.066; *P* < .001) (Figure 3).

**Figure 4.**
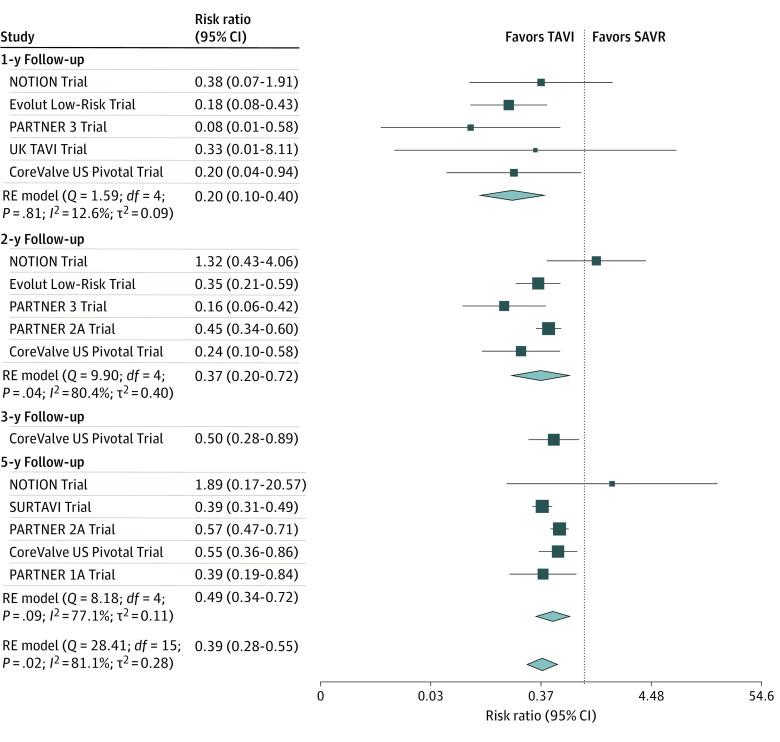
Forest Plot Presenting the Selective Risk of Loss to Follow-up There is a selective pattern characterized by a significant 2.56-fold lower risk of dropouts at follow-up for transcatheter aortic valve implantation (TAVI). The selective loss to follow-up appears to decrease with increasing follow-up time. The size of the solid squares is proportional to the weight of each study, the horizontal bars indicate the 95% CI for each study, the diamonds represent the pooled estimates with 95% CIs, and the vertical dotted line indicates a risk ratio equal to 1. NOTION indicates Nordic Aortic Valve Intervention Trial; PARTNER, Placement of Aortic Transcatheter Valve Trial; RE, random effect; and SAVR, surgical aortic valve replacement.

The analysis of selective loss to follow-up by follow-up years is depicted in Figure 4. The pooled RR of loss to follow-up in the TAVI group vs the SAVR group was 0.39 (95% CI, 0.28-0.55; *P* < .001), indicating that TAVI had a significant 2.56-fold lower risk of patients lost to follow-up. The selective loss to follow-up appears to decrease with increasing follow-up time, although meta-regression does not confirm the significance of this association (slope, 0.166; 95% CI, −0.034 to 0.366; *P* = .10) (eFigure 5 in Supplement 1).

### Analysis of the Proportion of Patients Who Received Additional Treatments

Data on additional procedures and additional revascularization are available for the CoreValve US Pivotal High Risk Trial, PARTNER 2A, SURTAVI, UK TAVI Trial, PARTNER 3, Evolut Low Risk Trial, and NOTION Trial.^2,3,4,5,6,7,8,10,11,12,13,15,16,36^

**Figure 5.**
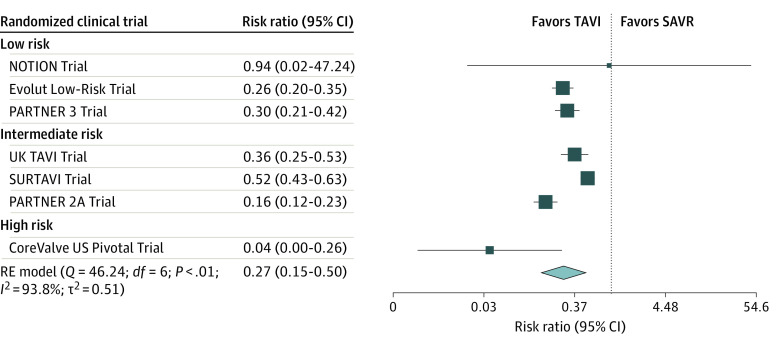
Forest Plot Presenting the Risk Ratio of Patients Who Received Additional Treatments in Transcatheter Aortic Valve Implantation (TAVI) vs Surgical Aortic Valve Replacement (SAVR) There is a selective pattern similar to deviation from assigned treatment and loss to follow-up, resulting in a 3.7-fold lower proportion of participants with additional procedures in the TAVI group. The size of the solid squares is proportional to the weight of each study, the horizontal bars indicate the 95% CI for each study, the diamond represents the pooled estimate with 95% CI, and the vertical dotted line indicates a risk ratio equal to 1. NOTION indicates Nordic Aortic Valve Intervention Trial; PARTNER, Placement of Aortic Transcatheter Valve Trial; RE, random effect; and SURTAVI, Surgical Replacement and Transcatheter Aortic Valve Implantation.

The pooled proportion of patients who underwent additional procedures was 10.4% (95% CI, 4.4%-18.5%) (eFigure 6 in Supplement 1). There was significant heterogeneity among studies (τ^2^ = 0.0241; *Q* test *P* < .001). There were differential rates of receipt of additional treatments in the 2 groups; the pooled proportion in the TAVI group was 4.6% (95% CI, 1.5%-9.3%) and in the SAVR group was 16.5% (95% CI, 7.5%-28.1%) (eFigure 7 in Supplement 1). This imbalance was statistically significant (RR, 0.27; 95% CI, 0.15-0.50; *P* < .001), indicating that TAVI has a 3.7-fold lower proportion of participants who undergo additional procedures (Figure 5). Only the NOTION Trial showed no significant difference between groups in the rate of patients who had additional procedures.

The pooled proportion of additional revascularizations was 7.5% (95% CI, 3.0%-13.9%), with significant heterogeneity among studies (τ^2^ = 0.0189; *Q* test *P* < .001) (eFigure 8 in Supplement 1). Again, there was a differential rate of additional myocardial revascularization in the 2 groups; the pooled proportion in the TAVI group was 4.5% (95% CI, 1.4%-9.0%) and in the SAVR group was 10.8% (95% CI, 4.8%-18.9%) (eFigure 9 in Supplement 1). The imbalance between groups was confirmed by the RR of patients with additional revascularization (RR, 0.40; 95% CI, 0.24-0.68; *P* < .001) (eFigure 10 in Supplement 1), indicating that TAVI has a 2.5-fold lower proportion of additional revascularization.

## Discussion

This study has 3 main findings. First, there are substantial proportions of DAT, loss to follow-up, and receipt of additional procedures in the seminal TAVI vs SAVR trials. Second, there is systematic imbalance in these parameters that trend in the same direction—with significantly lower proportions of DAT, loss to follow-up, and receipt of additional procedures in the TAVI groups. Therefore, despite randomization, there may remain biases that are critically associated with internal validity.

Third, attrition bias may be the most substantial threat to internal validity. Loss to follow-up threatens the ITT principle for guaranteeing an unbiased treatment comparison and should be minimized to preserve proper randomization. Although only 0% of losses ensures the benefit of randomization, 5% is the proposed cutoff for differentiating between little risk and intermediate risk of bias, while loss exceeding 20% could pose critical threats to validity.^19,22^ In the RCTs comparing TAVI and SAVR, there is a progressive increase of loss to follow-up reaching 5% at 2 years and 9% at 5 years that may progressively bias results with increasing follow-up time, resulting in noninformative data on durability and effectiveness at midterm and long term. The selective loss to follow-up is even more critical than the overall loss to follow-up, as it is not random^19^ and can potentially lead to informative censoring. Almost all the pivotal RCTs have a high imbalance of loss to follow-up, with a constant pattern favoring TAVI, reaching a loss of 10% in the TAVI groups and more than 20% in the SAVR groups at 5 years. This imbalance between treatments challenges internal validity. In addition, as the clinical events contributing to the primary outcomes (ie, death and stroke) are relatively rare in the study populations, the attrition bias introduced by significant loss to follow-up can be potentially amplified. Incompleteness of follow-up data is somehow inevitable; however, for minimizing incompleteness, the sample size should be estimated taking into account the length of follow-up and potential rates of loss, and the protocol should include methods for reducing and handling losses, including selection of centers with a history of excellent retention and use of statistical methods for analysis of missing data, such as imputation.^24^

Both the absolute rate of DAT and selective DAT can be associated with the ITT principle and undermine unbiased comparison groups at baseline.^20,21,22,24^ To obviate this risk, the Cochrane Rob 2 tool recommends estimating the effect of assignment to the intervention group by ITT analysis, which is a de facto standard for a clinical trial, and when as-treated analysis is performed.^37^ The use of ITT analysis, where all patients are counted according to their randomized group, ensures comparability between groups as obtained through randomization, maintains sample size, and eliminates bias. This approach measures both the efficacy of the intervention as assigned and overall adherence. In contrast, as-treated analysis refers to inclusion in the analysis of only patients who received the assigned intervention and per-protocol analysis refers to inclusion in the analysis of only patients who strictly adhered to the protocol. Although per-protocol analysis provides an estimate of the true efficacy of an intervention, it can be subject to bias as randomization has been violated. Poor trial quality (eg, due to poor protocol adherence or missing data) might bias results toward the null. In superiority studies, ITT analysis is therefore conservative, favoring the null hypothesis of no difference. However, in noninferiority trials (7 of 8 pivotal trials were designed as noninferiority trials with the NOTION Trial being the only superiority trial), where the alternative hypothesis is that of no difference, ITT analysis is no longer conservative as it can lead to false-positive results by favoring the alternative hypothesis. Consequently, noninferiority trials often consider both ITT and per-protocol analyses concurrently. Randomized clinical trials comparing TAVI and SAVR have chosen different approaches; nonetheless, in this case, the selective imbalance of DAT between TAVI and SAVR might raise concerns regarding internal validity in trials that primarily report as-treated or per-protocol analyses.

Performance bias is another critical issue in RCTs comparing TAVI and SAVR.^22^ Although most of the additional treatments are allowed by the protocol design and the formal definition of protocol deviation does not apply, selective application of additional procedures biases outcomes, as it expresses imbalanced baseline myocardial disease or systematic differences in the provided care. Myocardial revascularization strategies should be equally applicable to the 2 groups and patients requiring coronary revascularization should be included unless only surgical revascularization is considered appropriate,^36^ to avoid selective administration of adjunctive treatments. The high difference in rates of concomitant myocardial revascularization in TAVI groups vs SAVR groups is inconsistent with the equality of treatments; the trialists of PARTNER 2A acknowledge this point as they attribute such imbalanced rates to the undertreatment of significant coronary artery disease in the TAVI group.^4^

Similar considerations may be made with respect to the concomitant performance of thoracic aortic surgery or nonaortic valve procedures among patients undergoing SAVR; these procedures occurred at discrete, nonnegligible rates in PARTNER 3 and the Evolut Low Risk Trial. In other words, the administration of different therapeutic strategies to the same disease is in itself a selective protocol deviation with an intrinsic high risk of performance bias. The ESC/EACTS delegates interpreted the different proportion of myocardial revascularization in favor of SAVR and the different proportion of other additional procedures in favor of TAVI.^18^ This position is debatable based on the results of the ISCHEMIA (International Study of Comparative Health Effectiveness with Medical and Invasive Approaches) trial, which demonstrated that medical therapy holds a lower risk of adverse events in the short term while invasive treatment seems to yield a morbidity or mortality advantage only in the midterm and long term, confirming that each invasive maneuver holds an intrinsic risk of invasiveness-related adverse events.^38^ The more invasive the procedure, the higher the short-term risk of complications, while the potential advantage of an invasive treatment is likely to emerge in the midterm to long-term follow-up.^39,40^ As it is well expressed by EuroSCORE (the European System for Cardiac Operative Risk Evaluation) and STS PROM (Society of Thoracic Surgeons–Predicted Risk of Mortality) score, the adjunct of coronary artery bypass graft as well as other additional procedures significantly increases perioperative risk and represents an intrinsic baseline gap vs the isolated procedure, as also confirmed by the US Food and Drug Administration exploratory analysis of PARTNER 1A, where adding concomitant procedures to SAVR nearly doubled the risk of mortality (RR, 1.9; 95% CI, 1.3-2.7), biasing short-term results in favor of TAVI.^41^ The potential unfavorable effect of selective undertreatment reported in the TAVI groups will likely be unmasked only in the midterm, well beyond 1- to 2-year follow-up of the RCTs as evaluated in primary or secondary outcome measures.

The quantitative estimation of overall rates and selective imbalance of DAT, loss to follow-up, and receipt of additional procedures of our meta-analysis may help to revise at least some domains of the RoB 2 tool (eFigure 11 and eAppendix in Supplement 1). The imbalance of both proportion of additional procedures and myocardial revascularization favors TAVI as it increases the short-term risk from SAVR, while the beneficial association of a higher rate of myocardial revascularization may be evident in the long term. Hence, only the NOTION Trial is at low risk of performance bias, while the other 7 RCTs should be considered at high risk of performance bias. The high rate of exclusions after randomizations and the significant discrepancy between TAVI and SAVR suggest that PARTNER 1A, the CoreValve US Pivotal High Risk Trial, PARTNER 2A, PARTNER 3, SURTAVI, the UK TAVI Trial, and the Evolut Low Risk Trial are at high risk of attrition bias. These observations should lead clinicians to consider almost all RCTs comparing TAVI and SAVR to be at high risk of biases, and it might reduce their internal validity, although the direction of the biases may be only hypothesized without individual patient data.

### Limitations

This meta-analysis has some limitations. The main limitation is that we cannot predict how these imbalances act in the magnitude and direction of bias and overall benefit. Although it is described that selective rates of withdrawal may favor the treatment compared with the control, only analyses of individual patient data can confirm it. At this point of evidence, we can simply highlight that RCTs show serious methodological imbalances with a common selective pattern that can increase the risk of bias. The association of the risk of performance and attrition biases with trials’ primary outcomes is far beyond the aims of the present meta-analysis, which is focused on methodological issues and has been designed to quantify the key factors that might inflate performance and attrition biases. Only data sharing and an independent evaluation may overcome the existing limitations, permitting a deep understanding of the association between risk of bias and results.

## Conclusions

This systematic review and meta-analysis found that the RCT design does not protect from biases other than nonrandom allocation. In RCTs comparing TAVI vs SAVR, there were systematic imbalances in the proportion of DAT, loss to follow-up, and receipt of additional procedures and additional myocardial revascularization that can pose a serious threat to internal validity due to high risk of performance and attrition biases. The potential associations of this risk of bias with trials’ primary outcomes are beyond the aims of the present meta-analysis and need data sharing to be evaluated.

## Notes

Supplement 1. **eTable.** Updated Search Algorithms **eFigure 1.** Systematic Review—Flow Chart **eFigure 2.** Forest Plot Representing Pooled Proportion of Deviation From Assigned Treatment (DAT) **eFigure 3.** Forest Plot of Risk Ratio of Deviation From Assigned Treatment (DAT) in TAVI vs SAVR (Selective DAT) **eFigure 4.** Forest Plot Representing Pooled Proportion of Deviation From Assigned Treatment (DAT) in RCTs That Performed AT or Modified ITT Analysis **eFigure 5.** Meta-regression of Relationship Between RR of Loss to Follow-up in TAVI/SAVR and Follow-up Time **eFigure 6.** Forest Plot Representing Pooled Rate of Provided Additional Treatments **eFigure 7.** Forest Plot Representing Pooled Proportion of Provided Additional Treatments in TAVI and SAVR Groups **eFigure 8.** Forest Plot Representing Pooled Rate of Provided Additional Myocardial Revascularization **eFigure 9.** Forest Plot Representing Pooled Proportion of Provided Additional Myocardial Revascularization in TAVI and SAVR Groups **eFigure 10.** Forest Plot Presenting the Risk Ratio of Patients With Provided Additional Myocardial Revascularization in TAVI vs SAVR **eFigure 11.** Risk of Bias Evaluation Using the RoB 2.0 Tool **eAppendix.** Revised Cochrane Risk of Bias Tool for Randomized Trials (RoB 2 Tool) Comparing TAVI and SAVR

Supplement 2. **eTable.** Baseline Characteristics of the 8 Trials Included in the Meta-analysis

Supplement 3. **Nonauthor Collaborators.** International Evidence Grading Research Initiative Targeting Transparency and Quality (INTEGRITTY) Collaborators

Supplement 4.
Data Sharing Statement
