# Supplementary material for: Risk of Bias in Randomized Clinical Trials Comparing Transcatheter and Surgical Aortic Valve Replacement: A Systematic Review and Meta-analysis
Source: JAMA Netw Open. 2023 Jan 3;6(1):e2249321. doi: 10.1001/jamanetworkopen.2022.49321 (PMC9857525; doi:10.1001/jamanetworkopen.2022.49321)
Supplement: Supplement 1. — eTable. Updated Search Algorithms eFigure 1. Systematic Review—Flow Chart eFigure 2. Forest Plot Representing Pooled Proportion of Deviation From Assigned Treatment (DAT) eFigure 3. Forest Plot of Risk Ratio of Deviation From Assigned Treatment (DAT) in TAVI vs SAVR (Selective DAT) eFigure 4. Forest Plot Representing Pooled Proportion of Deviation From Assigned Treatment (DAT) in RCTs That Performed AT or Modified ITT Analysis eFigure 5. Meta-regression of Relationship Between RR of Loss to Follow-up in TAVI/SAVR and Follow-up Time eFigure 6. Forest Plot Representing Pooled Rate of Provided Additional Treatments eFigure 7. Forest Plot Representing Pooled Proportion of Provided Additional Treatments in TAVI and SAVR Groups eFigure 8. Forest Plot Representing Pooled Rate of Provided Additional Myocardial Revascularization eFigure 9. Forest Plot Representing Pooled Proportion of Provided Additional Myocardial Revascularization in TAVI and SAVR Groups eFigure 10. Forest Plot Presenting the Risk Ratio of Patients With Provided Additional Myocardial Revascularization in TAVI vs SAVR eFigure 11. Risk of Bias Evaluation Using the RoB 2.0 Tool eAppendix. Revised Cochrane Risk of Bias Tool for Randomized Trials (RoB 2 Tool) Comparing TAVI and SAVR [file jamanetwopen-e2249321-s001.pdf]

Supplementary Online Content

Barili F, Brophy JM, Ronco D, et al; International Evidence Grading Research Initiative Targeting Transparency and Quality (INTEGRITTY). Risk of bias in randomized clinical trials comparing transcatheter and surgical aortic valve replacement: a systematic review and meta-analysis. *JAMA Netw Open.* 2023;6(1):e2249321. doi:10.1001/jamanetworkopen.2022.49321

eTable. Updated Search Algorithms

eFigure 1. Systematic Review—Flow Chart

eFigure 2. Forest Plot Representing Pooled Proportion of Deviation From Assigned Treatment (DAT)

eFigure 3. Forest Plot of Risk Ratio of Deviation From Assigned Treatment (DAT) in TAVI vs SAVR (Selective DAT)

eFigure 4. Forest Plot Representing Pooled Proportion of Deviation From Assigned Treatment (DAT) in RCTs That Performed AT or Modified ITT Analysis

eFigure 5. Meta-regression of relationship Between RR of Loss to Follow-up in TAVI/SAVR and Follow-up Time

eFigure 6. Forest Plot Representing Pooled Rate of Provided Additional Treatments

eFigure 7. Forest Plot Representing Pooled Proportion of Provided Additional Treatments in TAVI and SAVR Groups

eFigure 8. Forest Plot Representing Pooled Rate of Provided Additional Myocardial Revascularization

eFigure 9. Forest Plot Representing Pooled Proportion of Provided Additional Myocardial Revascularization in TAVI and SAVR Groups

eFigure 10. Forest Plot Presenting the Risk Ratio of Patients With Provided Additional Myocardial Revascularization in TAVI vs SAVR

eFigure 11. Risk of Bias Evaluation Using the RoB 2.0 Tool

eAppendix. Revised Cochrane Risk of Bias Tool For Randomized Trials (RoB 2 Tool) Comparing TAVI and SAVR

This supplementary material has been provided by the authors to give readers additional information about their work.

© 2023 Barili F et al. *JAMA Network Open.*

eTable 1. Updated search algorithms (last search 06/06/2022).

**PUBMED**		
#1	("aortic valve stenosis/surgery"[MeSH Terms] OR "aortic valve insufficiency/surgery"[MeSH Terms] OR "surgical aortic valve replacement"[Title/Abstract] OR "surgical aortic valve replacement"[Other Term] OR "SAVR"[Title/Abstract])	25,037
#2	("Transcatheter Aortic Valve Replacement"[MeSH Terms] OR "trans-catheter aortic valve implantation"[Title/Abstract] OR "trans-catheter aortic valve implantation"[Other Term] OR "TAVI"[Title/Abstract] OR "TAVR"[Title/Abstract])	13,402
#3	("outcome*"[Title/Abstract] OR "follow-up"[Title/Abstract])	2,857,134
#4	("randomized controlled trial"[Publication Type] OR "trial"[Title/Abstract])	1,017,822
#5	#1 AND #2 AND #3 AND #4	**453**
**EMBASE**		
#1	('aortic regurgitation'/exp/mj OR 'aortic stenosis'/exp/mj OR 'surgical aortic valve replacement':ti,ab,kw OR savr:ti,ab,kw)	36,696
#2	('transcatheter aortic valve implantation'/exp OR 'trans-catheter aortic valve implantation':ti,ab,kw OR tavi:ti,ab,kw OR tavr:ti,ab,kw)	30,609
#3	'follow up':ab,ti OR outcome*:ab,ti	4,263,645
#4	trial:ti,ab,kw OR 'randomized controlled trial'/de	1,403,872
#5	#1 AND #2 AND #3 AND #4	549
#6	#5 AND ('controlled study'/de OR 'multicenter study'/de OR 'randomized controlled trial'/de)	**329**
**CENTRAL**		
#1	(surgical aortic valve replacement):ti,ab,kw OR (SAVR):ti,ab,kw	846
#2	(trans-catheter aortic valve implantation):ti,ab,kw OR (TAVI):ti,ab,kw OR (TVR):ti,ab,kw	1223
#3	#1 AND #2	**123**

© 2023 Barili F et al. *JAMA Network Open.*

eFigure 1. Systematic Review – Flow chart.

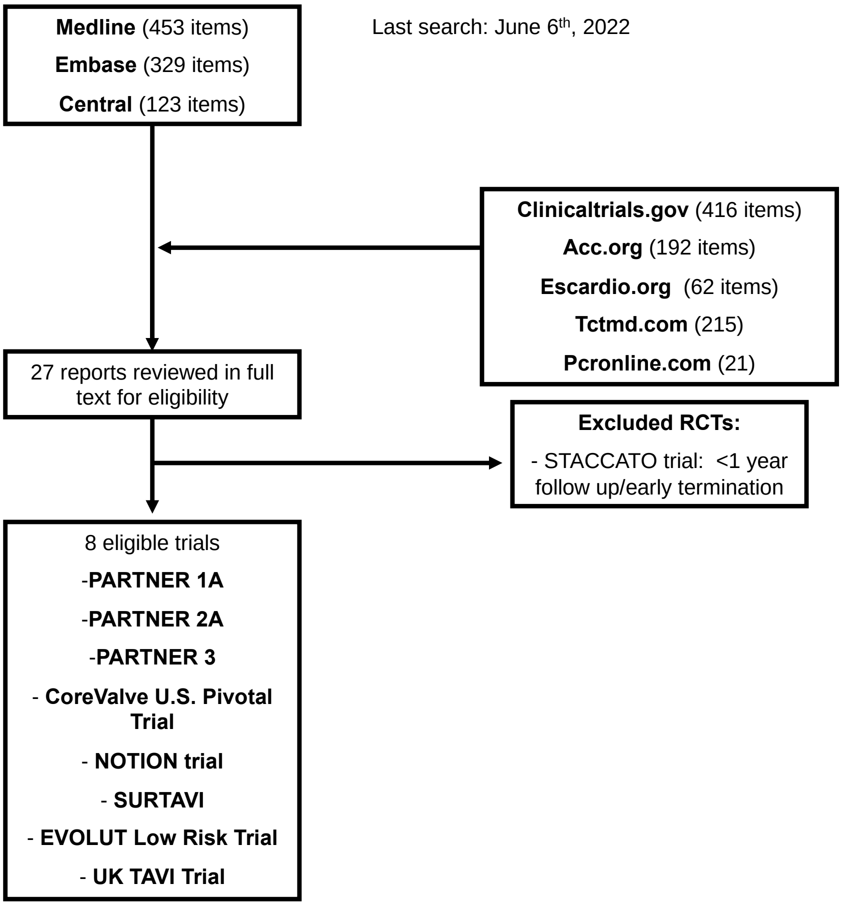

© 2023 Barili F et al. *JAMA Network Open.*

eFigure 2. Forest plot representing pooled proportion of deviation from assigned treatment (DAT).

Study	Events	Total	Weight	IV, Random, 95% CI
NOTION Trial	4	280	9.6%	0.014 [0.004; 0.036]
EVOLUT Low-Risk Trial	65	1468	13.3%	0.044 [0.034; 0.056]
PARTNER 3 Trial	50	1000	12.8%	0.050 [0.037; 0.065]
UK TAVI Trial	47	913	12.6%	0.051 [0.038; 0.068]
SURTAVI Trial	33	1660	13.5%	0.020 [0.014; 0.028]
PARTNER 2A Trial	94	2032	13.7%	0.046 [0.038; 0.056]
Corevalve US Pivotal Trial	47	797	12.4%	0.059 [0.044; 0.078]
PARTNER 1A Trial	42	699	12.1%	0.060 [0.044; 0.080]
**Total (95% CI)**		**8849**	**100.0%**	**0.042 [0.030; 0.056]**

Heterogeneity: $Tau^2 = 0.0017$; $Chi^2 = 49.28$, df = 7 ($P < 0.01$); $I^2 = 86\%$

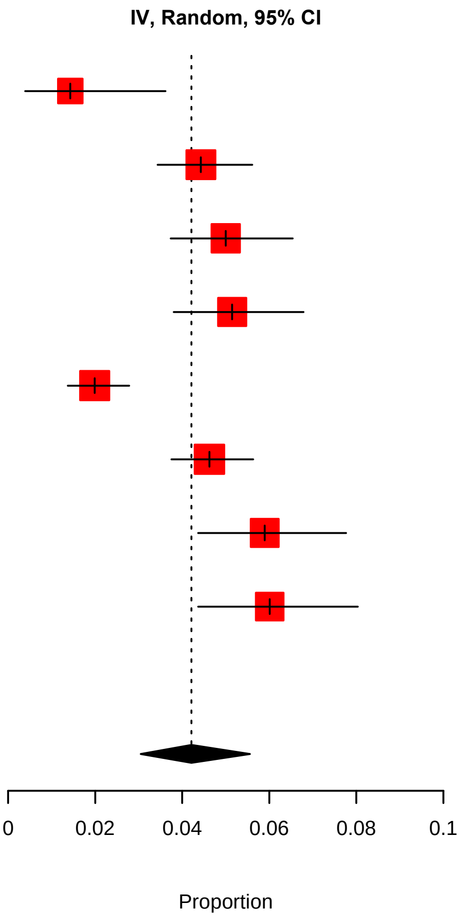

© 2023 Barili F et al. *JAMA Network Open.*

eFigure 3. Forest plot of Risk Ratio of Deviation from Assigned Treatment (DAT) in TAVI vs SAVR (selective DAT).

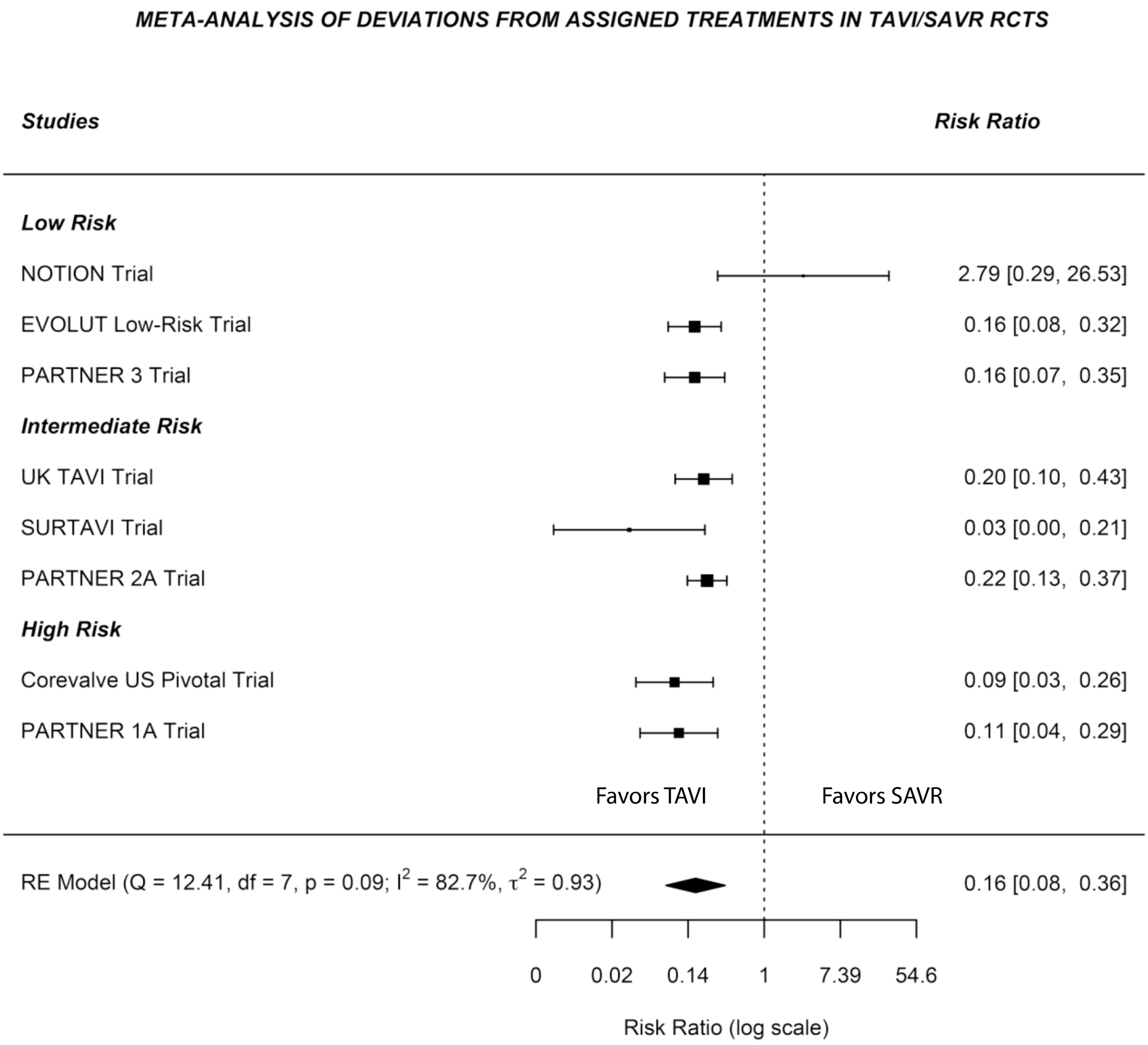

© 2023 Barili F et al. *JAMA Network Open.*

eFigure 4. Forest plot representing pooled proportion of deviation from assigned treatment (DAT) in RCTs that performed AT or modified ITT analysis.

Study	Events	Total	Weight	IV, Random, 95% CI
NOTION Trial	4	280	16.5%	0.014 [0.004; 0.036]
EVOLUT Low-Risk Trial	65	1468	21.3%	0.044 [0.034; 0.056]
PARTNER 3 Trial	50	1000	20.6%	0.050 [0.037; 0.065]
SURTAVI Trial	33	1660	21.4%	0.020 [0.014; 0.028]
Corevalve US Pivotal Trial	47	797	20.1%	0.059 [0.044; 0.078]
**Total (95% CI)**		**5205**	**100.0%**	**0.036 [0.021; 0.055]**

Heterogeneity: $Tau^2 = 0.0024$; $Chi^2 = 37.78$, df = 4 ($P < 0.01$); $I^2 = 89\%$

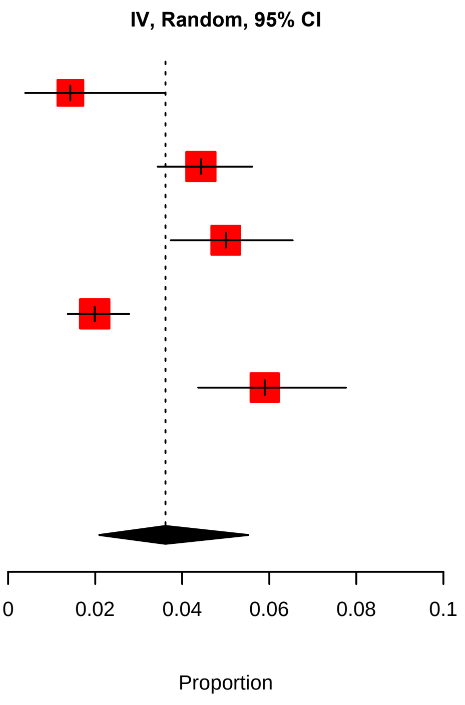

© 2023 Barili F et al. *JAMA Network Open.*

eFigure 5. Meta-regression of relationship between RR of loss to follow-up in TAVI/SAVR and follow-up time.

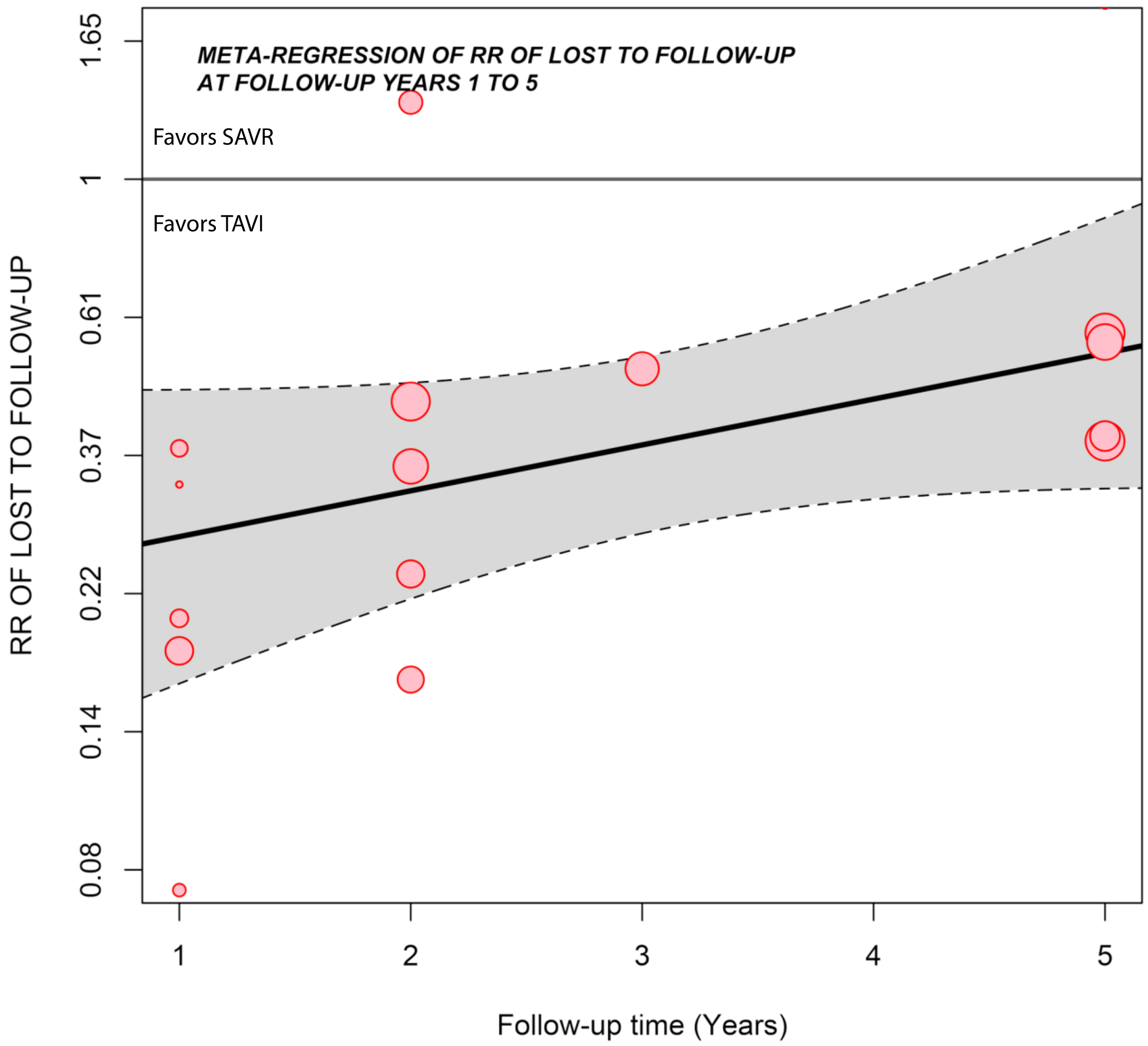

© 2023 Barili F et al. *JAMA Network Open.*

eFigure 6. Forest plot representing pooled rate of provided additional treatments.

Study	Events	Total	Weight	IV, Random, 95% CI
NOTION Trial	0	276	13.9%	0.000 [0.000; 0.013]
EVOLUT Low-Risk Trial	228	1403	14.4%	0.163 [0.144; 0.183]
PARTNER 3 Trial	159	950	14.3%	0.167 [0.144; 0.193]
UK TAVI Trial	123	913	14.3%	0.135 [0.113; 0.159]
SURTAVI Trial	346	1660	14.4%	0.208 [0.189; 0.229]
PARTNER 2A Trial	280	2032	14.4%	0.138 [0.123; 0.154]
Corevalve US Pivotal Trial	30	797	14.3%	0.038 [0.026; 0.053]
**Total (95% CI)**		**8031**	**100.0%**	**0.104 [0.044; 0.185]**

Heterogeneity: $Tau^2 = 0.0241$; $Chi^2 = 315.36$, df = 6 ($P < 0.01$); $I^2 = 98\%$

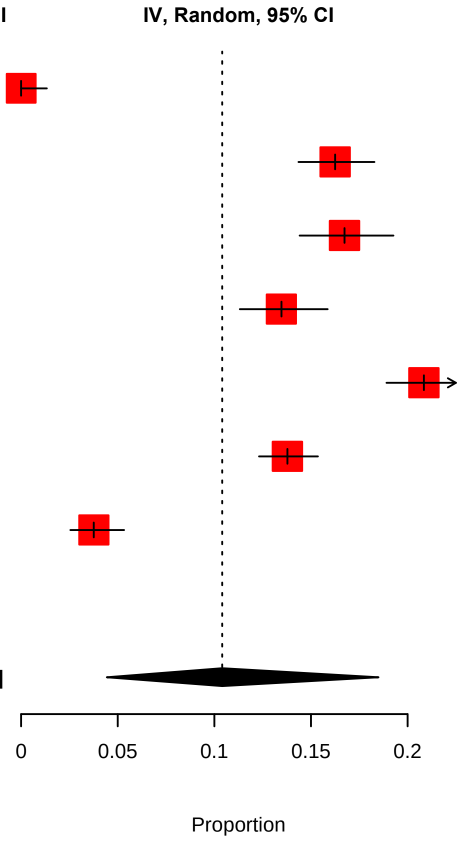

© 2023 Barili F et al. *JAMA Network Open.*

eFigure 7. Forest plot representing pooled proportion of provided additional treatments in TAVI and SAVR groups.

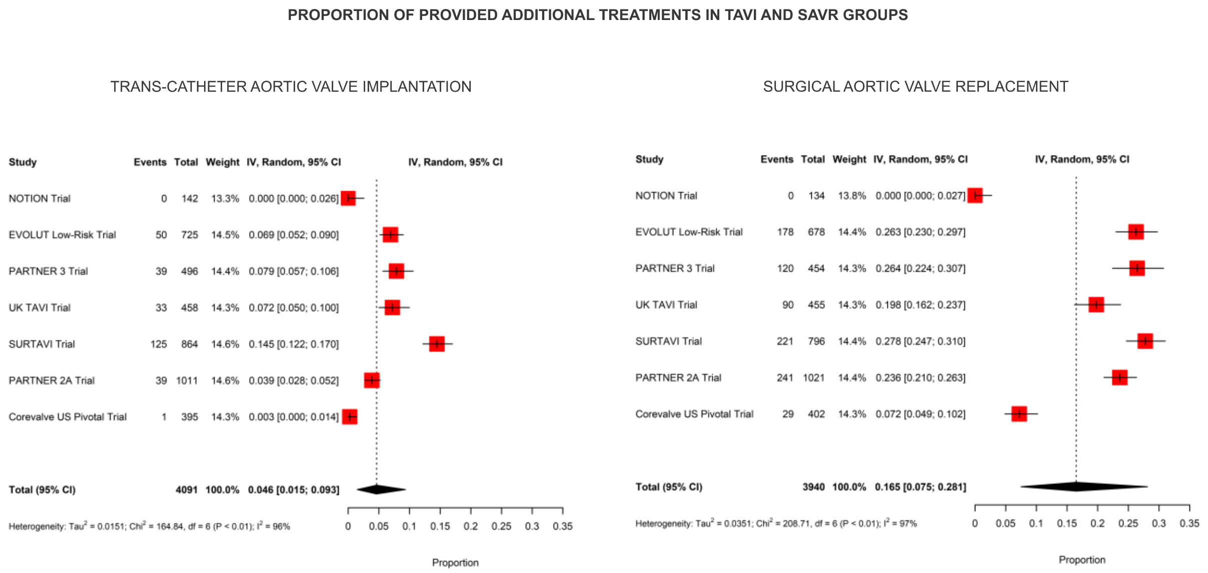

© 2023 Barili F et al. *JAMA Network Open.*

eFigure 8. Forest plot representing pooled rate of provided additional myocardial revascularization.

Study	Events	Total	Weight	IV, Random, 95% CI
NOTION Trial	0	276	13.9%	0.000 [0.000; 0.013]
EVOLUT Low-Risk Trial	142	1403	14.4%	0.101 [0.086; 0.118]
PARTNER 3 Trial	90	950	14.3%	0.095 [0.077; 0.115]
UK TAVI Trial	123	913	14.3%	0.135 [0.113; 0.159]
SURTAVI Trial	301	1660	14.4%	0.181 [0.163; 0.201]
PARTNER 2A Trial	187	2032	14.4%	0.092 [0.080; 0.105]
Corevalve US Pivotal Trial	18	797	14.3%	0.023 [0.013; 0.035]
**Total (95% CI)**		**8031**	**100.0%**	**0.075 [0.030; 0.139]**

Heterogeneity: $Tau^2 = 0.0189$; $Chi^2 = 290.80$, df = 6 ($P < 0.01$); $I^2 = 98\%$

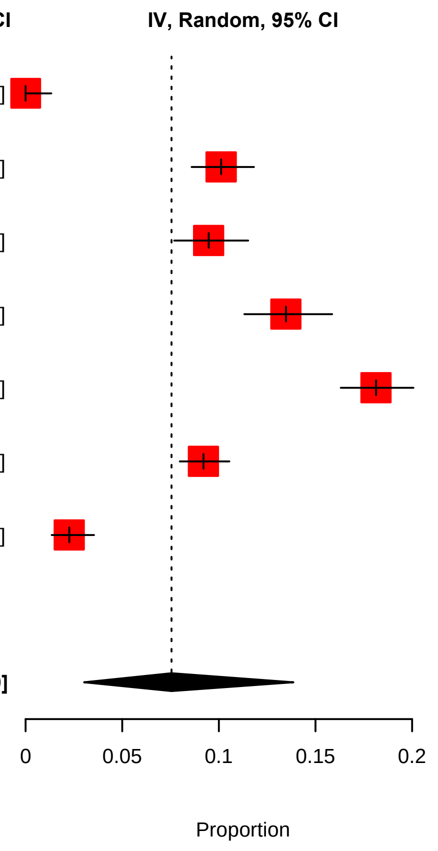

© 2023 Barili F et al. *JAMA Network Open.*

eFigure 9. Forest plot representing pooled proportion of provided additional myocardial revascularization in TAVI and SAVR groups.

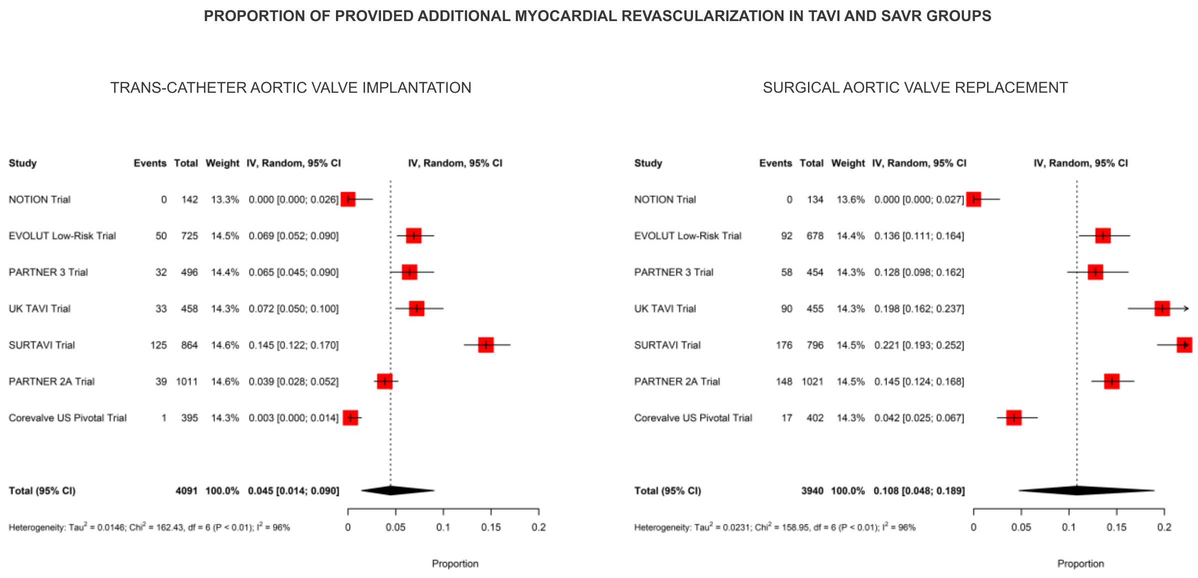

© 2023 Barili F et al. *JAMA Network Open.*

eFigure 10. Forest plot presenting the Risk Ratio of patients with provided additional myocardial revascularization in TAVI vs SAVR.

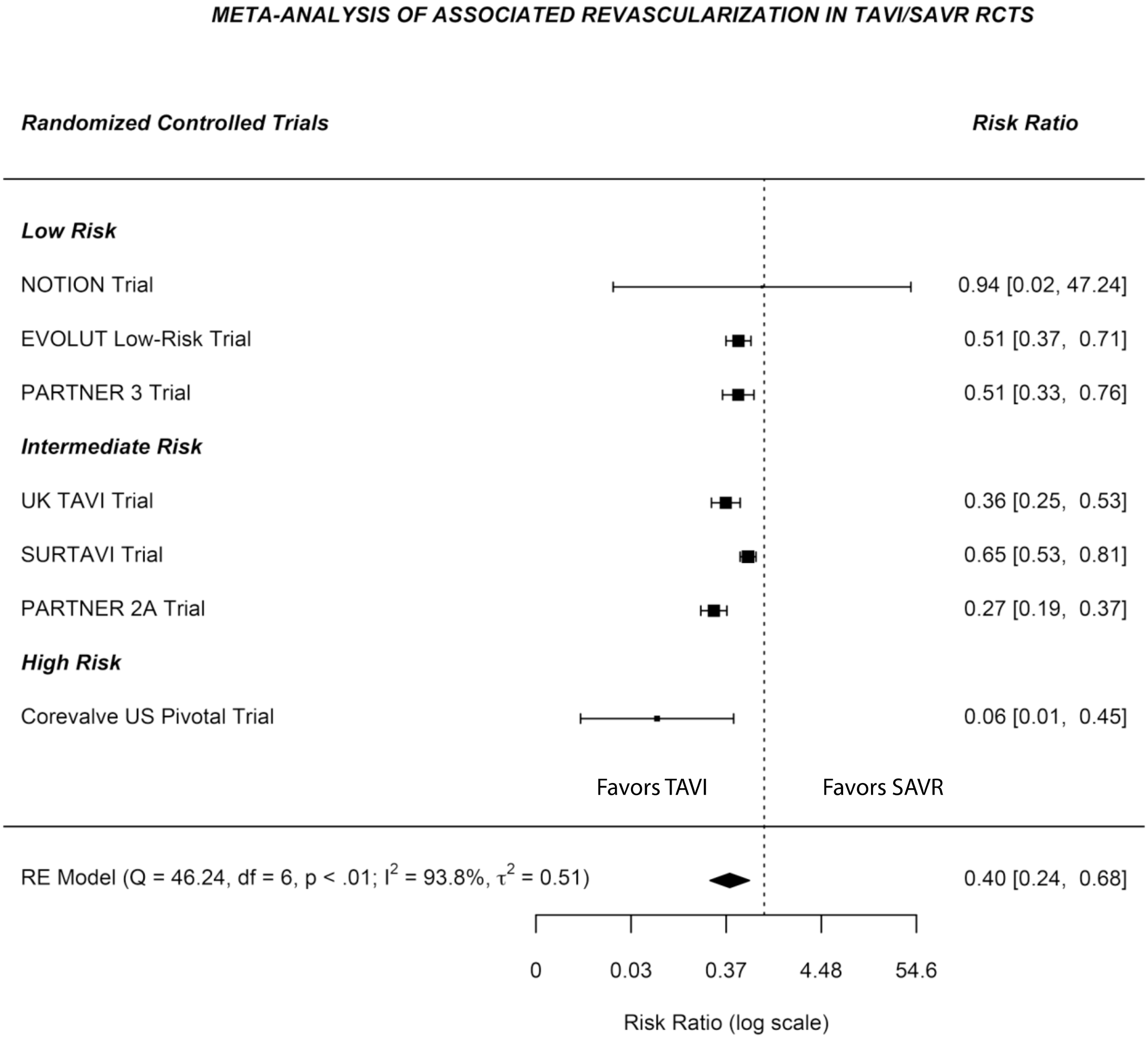

© 2023 Barili F et al. *JAMA Network Open.*

eFigure 11. Risk of bias evaluation using the RoB 2.0 tool.

	Randomization process	Deviations from intended interventions	Missing outcome data	Measurement of outcome	Selection of reported results	Overall
**PARTNER 1A**						
Non-inferiority primary outcome at 5 years	?	–	–	–	+	–
**CoreValve U.S. Pivotal High Risk**						
Non-inferiority primary outcome at 5 years	?	–	–	–	+	–
**PARTNER 2A**						
Non-inferiority primary outcome at 5 years	?	–	–	+	+	–
**SURTAVI**						
Non-inferiority primary outcome at 5 years	?	–	–	+	+	–
**UK TAVI**						
Non-inferiority primary outcome at 1 years	?	–	+	+	+	–
**PARTNER 3**						
Non-inferiority primary outcome at 2 years	?	–	+	+	+	–
**EVOLUT LR**						
Non-inferiority primary outcome at 2 years	?	–	+	+	+	–
**NOTION**						
Similarity primary outcome at 5 years	?	+	+	+	+	?

© 2023 Barili F et al. *JAMA Network Open.*

eAppendix. Revised Cochrane risk of bias tool for randomized trials (RoB 2 tool) comparing TAVI and SAVR [24]

General considerations.

The risk of bias among included trials performed in the ESC/EACTS 2021 Guidelines for Management of Heart Valve Disease by the four delegates [18] was updated by two researchers (FB and AP) with AA designated to solve potential disagreements using the RoB 2 tool [24] and incorporating also information provided by outcomes of this meta-analysis.

The quality assessment of eligible RCTs differs from the consensus document provided by the ESC/EACTS delegates [18] as PARTNER 2A and SURTAVI trials have updated 5-year follow-up and UK TAVI trial has been published after the development of the Document [24].

Five domains were assessed as required by RoB 2 [24]: randomization process, deviation from intended interventions, missing outcome data, measurement of outcome and selection of reported results. The single and overall risk of bias within each RCTs were assessed with the pre-specified algorithms and quantified as low risk of bias, some concerns and high risk of bias.

No disagreement between the two researchers emerged. The present summary discusses only domains with some grade of disagreement with the Consensus Document of the ESC/EACTS Guidelines [18]. Summarizing, the main difference in our assessment is related to Domain 2 (deviation from intended interventions), as we considered the imbalance of concomitant procedures/myocardial revascularization between groups relevant, in accordance with the Revised Cochrane risk of bias tool for randomized trials. In the RoB 2 tool, relevant co-interventions are the interventions or exposures that individuals might receive with or after starting the intervention of interest, which are related to the intervention received and which are prognostic for the outcome of interest. As describe in RoB 2, the study results are at risk of bias when the co-interventions are not balanced between intervention groups and were likely to impact on the outcome. Concomitant procedures and myocardial revascularization meet all these criteria. The evaluation in Domain 3

© 2023 Barili F et al. *JAMA Network Open.*

(missing outcome data) differs substantially for RCTs with follow-up updated to 5-year, as 5-year follow-up is related to a significant increase of missing to a rate over 15-20% at least in one group, defined as "high".

PARTNER 1A (Non-inferiority primary outcome at 5 years).

Domain 1 (Randomization Process). The researchers agree with the ESC/EACTS delegates that some concerns exist for the lack of description of a central randomization process.

Domain 2 (Deviation from Intended Intervention). There is agreement with ESC/EACTS guidelines evaluation. There is a significant selective rate of DAT (RR 0.11, 95%CI 0.04-029), hence deviation from intended intervention are unbalanced between groups. No information on concomitant procedures/concomitant are available. The trial is at high risk of bias for Domain 2.

Domain 3 (Missing outcome data). There is agreement with ESC/EACTS guidelines evaluation. Although the proportion at follow-up is low (4.6%, 95%CI 3.2-6.4%), there is a significant imbalance of the number of loss to follow-up at five years between (RR 0.39 95%CI 0.19-0.84). The trial is at high risk of bias for Domain 3.

Domain 4 (Measurement of outcome). There is agreement with ESC/EACTS guidelines evaluation, interpretation of results needs to take into account the clear access route by treatment interaction. The trial is at high risk of bias for Domain 4.

Domain 5. There is agreement with ESC/EACTS guidelines evaluation, the trial is at low risk of bias for Domain 5.

Overall. Overall the Partner 1A is at high risk of bias regarding non-inferiority at 5 years, as it is judged to be at high risk of bias in 3 domains (2 to 4).

© 2023 Barili F et al. *JAMA Network Open.*

COREVALVE US PIVOTAL TRIAL (Non-inferiority primary outcome at 5 years).

Domain 1 (Randomization Process). The researchers agree with the EACTS delegates that some concerns exist as stratification by site and blocking with randomly varied block sizes is related to a theoretical risk of selection bias of unclear magnitude and direction. The trial raises some concerns in Domain 1.

Domain 2 (Deviation from Intended Intervention). There was agreement with ESC/EACTS delegates. A substantial proportion of patients did not undergo the intended procedure and our meta-analysis confirms significant unbalanced deviation from intended intervention between groups (RR 0.09, 95%CI 0.03-0.26). Also a significant imbalance in the proportion of concomitant procedures (RR 0.04 95%CI 0.00-0.26) and concomitant myocardial revascularization (RR 0.06, 95%CI 0.01-0.45) is evident, although the overall proportion of concomitant procedures and myocardial revascularization is low (2.6% and 2.6% respectively). The trial is at high risk of bias for Domain 2.

Domain 3 (Missing outcome data). There is agreement with ESC/EACTS guidelines evaluation. There is a high proportion of lost-to-follow up at 5 years (10.3%, 95%CI 8.2%-12.7%) with a significant imbalance between groups (RR 0.39 95%CI 0.19-0.84). The trial is at high risk of bias for Domain 3.

Domain 4 (Measurement of outcome). There is agreement with ESC/EACTS guidelines evaluation, interpretation of results needs to take into account the clear access route by treatment interaction. The trial is at high risk of bias for Domain 4.

Domain 5. There is agreement with ESC/EACTS guidelines evaluation, the trial is at low risk of bias for Domain 5.

Overall. Overall the Corevalve US Pivotal Trial is at high risk of bias regarding non-inferiority at 5 years, as it is judged to be at high risk of bias in 3 domains (2 to 4).

PARTNER 2A TRIAL (Non-inferiority primary outcome at 5 years).

© 2023 Barili F et al. *JAMA Network Open.*

Domain 1 (Randomization Process). We agree with the EACTS delegates of the European Guidelines that some concerns exist as stratification by site and blocking with randomly varied block sizes is related to a theoretical risk of selection bias of unclear magnitude and direction. The trial raises some concerns in Domain 1.

Domain 2 (Deviation from Intended Intervention). We do not agree with ESC/EACTS delegates. Our meta-analysis confirmed significant imbalances similar to PARTNER1A or Corevalve US Pivotal Trial. The proportion of DAT is 4.6% and there is a significant selective rate of DAT (RR 0.22, 95%CI 0.13-0.37), meaning an unbalanced deviation from intended intervention between groups. Also a significant imbalance in the proportion of concomitant procedures (3.9% in TAVI, 14.6% in SAVR; RR 0.16 95%CI 0.12-0.23) and concomitant myocardial revascularization (RR 0.27, 95%CI 0.19-0.37) was evident. As explained in the Discussion, the potential advantages of TAVI (lower perioperative risk related to lower proportion of associated procedures) and SAVR (protective effect of higher proportion of myocardial revascularization) happen in different time-span; as clearly showed by evidence, the positive effect of higher proportion of myocardial revascularization will be evident in the mid and long-term time, beyond the existing available follow-up and it does not balance the actual perioperative risk intrinsically held by associated procedures/myocardial revascularization. The trial is at high risk of bias for Domain 2.

Domain 3 (Missing outcome data). Our judgement is not aligned with ESC/EACTS delegates; our different evaluation is related to the different available follow-up, being updated from 2 years to 5 years. There was a high proportion of lost to follow-up at 5 years (16.0%, 95%CI 14.5%-17.7%) with a significant imbalance between groups (RR 0.57 95%CI 0.47-0.71). The trial is at high risk of bias for Domain 3.

Domain 4 (Measurement of outcome). There is agreement with ESC/EACTS guidelines evaluation. The trial is at low risk of bias for Domain 4.

Domain 5 (Selection of reported results). There is agreement with ESC/EACTS guidelines evaluation, the trial is at low risk of bias for Domain 5.

© 2023 Barili F et al. *JAMA Network Open.*

Overall. Overall the PARTNER 2A Trial is at high risk of bias regarding non-inferiority at 5 years, as it is judged to be at high risk of bias in 2 domains (2 and 3).

SURTAVI TRIAL (Non-inferiority primary outcome at 5 years).

Domain 1 (Randomization Process). We agree with the EACTS delegates of the European Guidelines that some concerns exist as stratification by site and blocking with randomly varied block sizes is related to a theoretical risk of selection bias of unclear magnitude and direction. The trial raises some concerns in Domain 1.

Domain 2 (Deviation from Intended Intervention). There's no agreement with ESC/EACTS delegates. Our meta-analysis confirms significant imbalances similar to PARTNER1A, Corevalve US Pivotal Trial and PARTNER 2A. There is a significant selective rate of DAT (RR 0.03, 95%CI 0.00-0.21), meaning an unbalanced deviation from intended intervention between groups. Also a significant imbalance in the proportion of concomitant procedures (RR 0.52 95%CI 0.43-0.63) and concomitant myocardial revascularization (RR 0.65, 95%CI 0.53-0.81) between groups is evident. As for PARTNER 2A, the potential advantages of TAVI (lower perioperative risk related to lower proportion of associated procedures) and SAVR (protective effect of higher proportion of myocardial revascularization) happen in different time-span; as clearly showed by evidence, the positive effect of higher proportion of myocardial revascularization will be evident in the mid and long-term time, beyond the existing available follow-up and it does not balance the actual perioperative risk intrinsically held by associated procedures. The trial is at high risk of bias for Domain 2.

Domain 3 (Missing outcome data). Our judgement is not aligned with ESC/EACTS delegates; our different evaluation is related to the different available follow-up, being updated from 2 years to 5 years. There was a high proportion of lost-to-follow up at 5 years (18.3%, 95%CI 16.5%-20.3%). Moreover the proportion of missing at 5 years are highly different between groups with a significant imbalance (RR 0.39, 95%CI 0.31-0.49). The trial is at high risk of bias for Domain 3.

© 2023 Barili F et al. *JAMA Network Open.*

Domain 4 (Measurement of outcome). There was agreement with ESC/EACTS guidelines evaluation. The trial is at low risk of bias for Domain 4.

Domain 5 (Selection of reported results). There was agreement with ESC/EACTS guidelines evaluation, the trial is at low risk of bias for Domain 5.

Overall. Overall the SURTAVI Trial is at high risk of bias regarding non-inferiority at 5 years, as it is judged to be at high risk of bias in 2 domains (2 and 3).

NOTION TRIAL (Non-inferiority primary outcome at 5 years).

Domain 1 (Randomization Process). We agree with the EACTS delegates of the European Guidelines that some concerns exist as stratification by site and blocking with randomly varied block sizes is related to a theoretical risk of selection bias of unclear magnitude and direction. The trial raises some concerns in Domain 1.

Domain 2 (Deviation from Intended Intervention). There is agreement with ESC/EACTS delegates. The proportion of DAT is low (2%) with no significant differences between treatments (RR 2.79, 95CI 0.29-26.53). There are no associated procedures, hence outcomes are not potentially influenced. The trial is at low risk of bias for Domain 2.

Domain 3 (Missing outcome data). There is agreement with ESC/EACTS delegates. The proportion of missing at 5 years is very low (1.1%) with no imbalances between group (RR 1.89, 95%CI 0.17-20.57). The trial is at low risk of bias for Domain 3.

Domain 4 (Measurement of outcome). There is agreement with ESC/EACTS delegates. The trial is at low risk of bias for Domain 4.

Domain 5 (Selection of reported results). There was agreement with ESC/EACTS guidelines evaluation, the trial is at low risk of bias for Domain 5.

© 2023 Barili F et al. *JAMA Network Open.*

Overall. Overall the NOTION Trial raises some concerns in the risk-of-bias judgement, as it raises some concerns in Domain 1.

PARTNER 3 TRIAL (Non-inferiority primary outcome at 2 years).

Domain 1 (Randomization Process). We agree with the EACTS delegates of the European Guidelines that some concerns exist as stratification by site and blocking with randomly varied block sizes is related to a theoretical risk of selection bias of unclear magnitude and direction. The trial raises some concerns in Domain 1.

Domain 2 (Deviation from Intended Intervention). We do not agree with ESC/EACTS delegates. Our meta-analysis confirmed significant imbalances similar to RCTs in high and mid-risk. The proportion of DAT is 5% with a significant imbalance between groups (RR 0.16, 95%CI 0.07-0.35). There is a high proportion of associated procedures (16.7%) and concomitant myocardial revascularization (9.5%) with a significant imbalance in the proportion between groups (Associated procedures: 7.9% in TAVI, 26.4% in SAVR; RR 0.30 95%CI 0.21-0.42. Concomitant myocardial revascularization: RR 0.51, 95%CI 0.33-0.76). The positive effect of higher proportion of myocardial revascularization will be evident in the mid and long-term time, beyond the existing available follow-up and it does not balance the actual perioperative risk intrinsically held by associated procedures/myocardial revascularization. The trial is at high risk of bias for Domain 2.

Domain 3 (Missing outcome data). There is agreement with ESC/EACTS delegates. Although there is a significant imbalance of loss to follow-up at 2 years between groups (RR 0.16, 95%CI 0.06-0.42), the overall proportion of missing is low (3.5%), mitigating the differences between treatments. The trial is at low risk of bias for Domain 3.

Domain 4 (Measurement of outcome). There was agreement with ESC/EACTS guidelines evaluation. The trial is at low risk of bias for Domain 4.

© 2023 Barili F et al. *JAMA Network Open.*

Domain 5 (Selection of reported results). There was agreement with ESC/EACTS guidelines evaluation, the trial is at low risk of bias for Domain 5.

Overall. Overall the PARTNER 3 Trial is at high risk of bias regarding non-inferiority at 2 years, as it is judged to be at high risk of bias in domain 2.

EVOLUT LOW-RISK TRIAL (Non-inferiority primary outcome at 2 years).

Domain 1 (Randomization Process). We agree with the EACTS delegates of the European Guidelines that some concerns exist as stratification by site and blocking with randomly varied block sizes is related to a theoretical risk of selection bias of unclear magnitude and direction. The trial raises some concerns in Domain 1.

Domain 2 (Deviation from Intended Intervention). We do not agree with ESC/EACTS delegates. Our meta-analysis confirmed significant imbalances similar to RCTs in high and mid-risk. There is a significant selective rate of DAT (RR 0.16, 95%CI 0.08-0.32), meaning an unbalanced deviation from intended intervention between groups. There is a high proportion of associated procedures (16.3%) and concomitant myocardial revascularization (10.1%) with a significant imbalance in the proportion between groups (Associated procedures: RR 0.26 95%CI 0.20-0.35. Concomitant myocardial revascularization: RR 0.51, 95%CI 0.37-0.71). The positive effect of higher proportion of myocardial revascularization will be evident in the mid and long-term time, beyond the existing available follow-up and it does not balance the actual perioperative risk intrinsically held by associated procedures. The trial is at high risk of bias for Domain 2.

Domain 3 (Missing outcome data). We agree with ESC/EACTS delegates. Although there is a significant imbalance of the number of loss to follow-up at 2 years between groups (RR 0.35, 95%CI 0.21-0.59), the overall proportion of missing is low (5.2%), mitigating the differences between treatments. The trial is at low risk of bias for Domain 3.

© 2023 Barili F et al. *JAMA Network Open.*

Domain 4 (Measurement of outcome). There was agreement with ESC/EACTS guidelines evaluation. The trial is at low risk of bias for Domain 4.

Domain 5 (Selection of reported results). There was agreement with ESC/EACTS guidelines evaluation, the trial is at low risk of bias for Domain 5.

Overall. Overall the EVOLUT Low Risk Trial is at high risk of bias regarding non-inferiority at 2 years, as it is judged to be at high risk of bias in domain 2.

UK TAVI Trial (Non-inferiority primary outcome at 1 years).

Domain 1 (Randomization Process). Some concerns exist as, from a statistical point of view, minimization it does not satisfy randomness, which is the basic assumption of statistical inference leading to a theoretical risk of selection bias of unclear magnitude and direction. The trial raises some concerns in Domain 1.

Domain 2 (Deviation from Intended Intervention). There is a significant selective rate of DAT (RR 0.20, 95%CI 0.10-0.43), meaning an unbalanced deviation from intended intervention between groups. Also a significant imbalance in the proportion of concomitant myocardial revascularization (RR 0.36, 95%CI 0.25-0.53) was evident. The trial is at high risk of bias for Domain 2.

Domain 3 (Missing outcome data). The trial is at low risk of bias for Domain 3.

Domain 4 (Measurement of outcome). The trial is at low risk of bias for Domain 4.

Domain 5 (Selection of reported results). The trial is at low risk of bias for Domain 5.

Overall. Overall the UK Trial is at high risk of bias regarding non-inferiority at 1 years, as it is judged to be at high risk of bias in domain 2.

© 2023 Barili F et al. *JAMA Network Open.*
