# Supplementary material for: Risk of Bias in Randomized Clinical Trials Comparing Transcatheter and Surgical Aortic Valve Replacement: A Systematic Review and Meta-analysis
Source: JAMA Netw Open. 2023 Jan 3;6(1):e2249321. doi: 10.1001/jamanetworkopen.2022.49321 (PMC9857525; doi:10.1001/jamanetworkopen.2022.49321)
Supplement: Supplement 3. — Nonauthor Collaborators. International Evidence Grading Research Initiative Targeting Transparency and Quality (INTEGRITTY) Collaborators [file jamanetwopen-e2249321-s003.pdf]

Supplemental Online Content: Nonauthor Collaborators
*First name, last name, and suffix (if applicable) are required and will appear in PubMed.

*Group Name(s): International Evidence Grading Research Initiative Targeting Transparency and Data Quality (INTEGRITTY)							
***First Name and Middle Initial(s)**	***Last Name**	***Suffix (eg, Jr, III)**	Academic Degrees	Institution	Location (city, state/province, country)	Role or Contribution, eg, chair, principal investigator	Group (if more than 1 Group listed in the byline) and/or Subgroup (eg, Steering Committee)
Raffaele	De Caterina		MD Prof	*University Cardiology Division Univers*	Pisa, Italy	Founder member	
Rafael	Sadaba		MD	*Department of Cardiac Surgery, Hosp*	Navarra, Spain	Founder member	
Martin	Misfeld		MD Prof	*University Department for Cardiac Su*	Leipzig, Germany	Founder member	
Milan	Milojevic		MD	*Department of Cardiac Surgery and C*	*Belgrade, Serbia*	Founder member	
William	Boden		MD	*Boston University School of Medicine*	Boston, US.	Founder member	
Arthur	Albuquerque		MD	*School of Medicine, Universidade Fed*	Rio de Janeiro, Brasil	Founder member	
Walter	Gomes		MD	*Cardiovascular Surgery, Pirajussara H*	*Sao Paulo, Brasil*	Founder member	
Victor	Dayan		MD	*Centro Cardiovascular Universitario, U*	Uruguay	Founder member	
Ovidio A.	García-Villarreal		MD	*Mexican College of Cardiovascular an*	*Mexico City, México*	Founder member	
Jorge	Rodriguez-Roda Stuart		MD	Chief, Servicio de Cirugía Cardiovascu	Madrid, Spain	Founder member	
