# Supplementary material for: Risk of Bias in Randomized Clinical Trials Comparing Transcatheter and Surgical Aortic Valve Replacement: A Systematic Review and Meta-analysis
Source: JAMA Netw Open. 2023 Jan 3;6(1):e2249321. doi: 10.1001/jamanetworkopen.2022.49321 (PMC9857525; doi:10.1001/jamanetworkopen.2022.49321)
Supplement: Supplement 4. — Data Sharing Statement [file jamanetwopen-e2249321-s004.pdf]

Data Sharing Statement

Barili. Risk of Bias in Randomized Clinical Trials Comparing Transcatheter and Surgical Aortic Valve Replacement. *JAMA Netw Open*. Published January 03, 2023.
doi:10.1001/jamanetworkopen.2022.49321

Data
Data available: No

Additional Information
Explanation for why data not available: Data underlying the meta-analysis are retrieved from published randomized clinical trials and hence already available in literature; no unpublished data were employed. However, the collected data underlying this article will be shared on reasonable request to the corresponding author.
